# Detection of Early Age-Related Macular Degeneration Using Novel Functional Parameters of the Focal Cone Electroretinogram

**DOI:** 10.1371/journal.pone.0096742

**Published:** 2014-05-05

**Authors:** Ashley Wood, Thomas Margrain, Alison Mary Binns

**Affiliations:** 1 School of Optometry and Vision Sciences, Cardiff University, Cardiff, United Kingdom; 2 Division of Optometry and Visual Science, School of Health Sciences, City University London, London, United Kingdom; Medical University of South Carolina, United States of America

## Abstract

The focal cone electroretinogram is a sensitive marker for macular disease, but have we unlocked its full potential? Typically assessment of waveform parameters is subjective and focuses on a small number of locations (e.g. the a-wave). This study evaluated the discriminatory and diagnostic potential of 4 conventional and 15 novel, objectively determined, parameters in patients with early Age-related Macular Degeneration. Focal cone electroretinograms were recorded in 54 participants with early Age-related Macular Degeneration (72.9±8.2 years) and 54 healthy controls (69±7.7 years). Conventional a and b wave amplitudes and implicit times were measured and compared to novel parameters derived from both the 1st and 2nd derivatives and the frequency-domain power spectrum of the electroretinogram.Statistically significant differences between groups were shown for all conventional parameters, the majority of 1st and 2nd derivative parameters and the power spectrum at 25 and 30 Hz. Receiver operating characteristics showed that both conventional and 1st and 2nd derivative implicit times had provided the best diagnostic potential. A regression model showed a small improvement over any individual parameter investigated. The non-conventional parameters enhanced the objective evaluation of the focal electroretinogram, especially when the amplitude was low. Furthermore, the novel parameters described here allow the implicit time of the electroretinogram to be probed at points other than the peaks of the a and b waves. Consequently these novel analysis techniques could prove valuable in future electrophysiological investigation, detection and monitoring of Age-related Macular Degeneration.

## Introduction

Age-related Macular Degeneration (AMD) is the leading cause of irreversible vision loss in the western world and accounts for over 50% of all sight impairment registrations in the United Kingdom [1]. The prevalence of AMD is expected to increase globally over the next 40 years due to a predicted 3-fold increase in the number of people over 60 years of age [2]. However, effective treatments (e.g. anti-VEGF therapy) are currently only available for the neovascular "wet" subtype of the condition which accounts for about 10% of cases [3].

In recent years the understanding of the pathological processes underlying AMD disease progression has greatly improved [4]–[6]. As a consequence, an increasing number of mechanisms have been identified as possible targets for treatment development, particularly in the early stages of disease [4]. The need, therefore, for sensitive, effective and ideally objective measures of retinal function [7], to evaluate these potential interventions in patients with early AMD, may never have been greater.

The electroretinogram (ERG) provides a quantitative and almost uniquely objective measure of retinal function. The light evoked ERG waveform is a summed bio-electrical potential comprising the contributions of many different intra-retinal processes. Components of the waveform, such as the “a” and “b” wave have been attributed to specific retinal origins [8], [9]. Measured changes in timing (implicit time) or magnitude (amplitude) of these components reflect underlying changes in retinal function and have been shown to be sensitive across a range of retinal pathologies [10]–[12]. Although the conventional full-field ERG is not sensitive to early AMD [13], [14], focal ERG techniques stimulating only the central region of the retina have been shown to be sensitive to AMD [15]–[23]. For example, the focal cone ERG has been shown to be abnormal in early AMD [17], [21], neovascular AMD [16], and dry AMD [19], with deficits showing a correlation with the severity of fundus changes [19], [20], [23], and a potential prognostic ability to predict individuals who will convert from early to advanced AMD [22]. However, a question remains over what is the best way of quantifying the elicited ERG waveforms.

**Figure 1:**
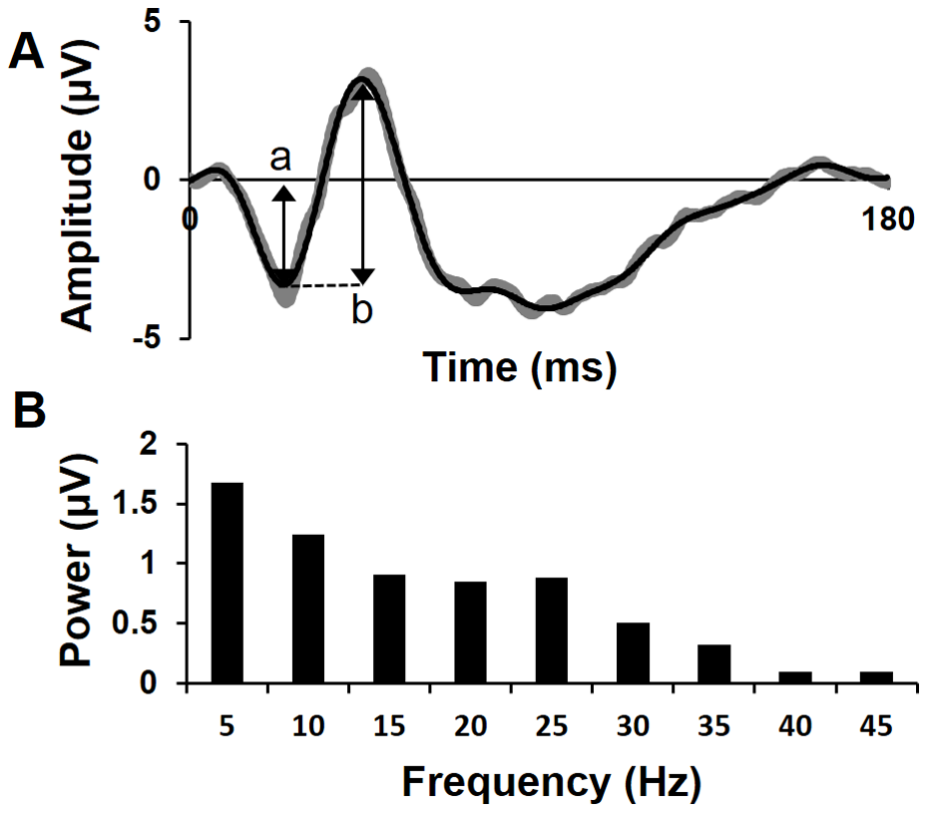
Example focal cone ERG waveform and frequency-domain power spectrum. (A) A focal cone ERG waveform before (shown in grey) and after fourier smoothing (overlaid in black). Arrows exemplify measurement of ‘a’ and ‘b’ wave amplitudes. (B) The focal cone ERG shown in the frequency-domain (power spectrum) at the fundamental frequency (f_0_ = 5 Hz) and its harmonics up to 45 Hz.

**Figure 2:**
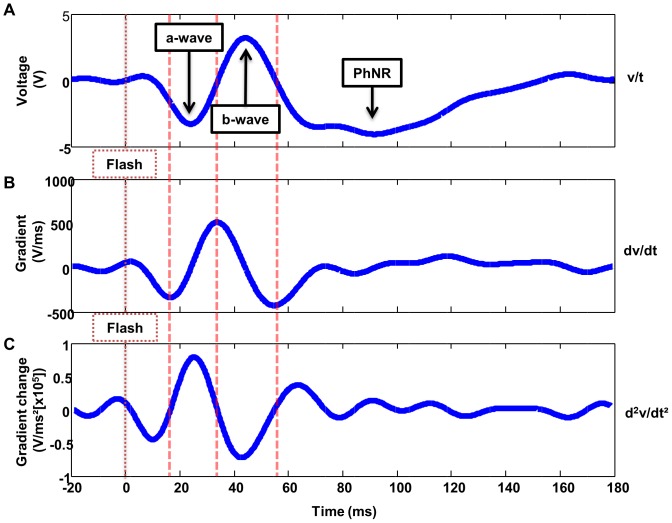
Labelled focal cone ERG waveform and derivatives. (A) A fourier smoothed focal cone ERG waveform with 20 ms pre-flash baseline and the conventional waveform components, the negative a-wave, the positive b wave and the Photopic Negative Response (PhNR) labelled. (B) 1^st^ derivative showing gradient or the “rate of change” against time. (C) 2^nd^ derivative showing zero crossings which correspond to inflection points in the original waveform. Dashed vertical lines correspond to the location of 3 inflection points in the Fourier smoothed waveform (A) and time of flash (labelled) across the 3 waveforms (A, B & C) shown.

Clinically, the interpretation of the ERG has focused on the measurement of prominent and easily identifiable waveform features in the time-domain (voltage against time) which some have, possibly unfairly, referred to as “bumpology”. Essentially, this approach involves the measurement of the amplitude and implicit time of the most prominent peaks and troughs within the waveform, most commonly the a and b waves (see Figures 1 & 2). This is often a subjective method, which relies on visual inspection of the ERG waveform to identify maxima and minima, whilst attempting to disregard any peaks which are likely to be attributable to noise. The subjectivity of this approach becomes more of a concern in the assessment of the focal ERG, where the signal-to-noise ratio is much lower than for the full-field response. Furthermore, the conventionally used reference points, such as the a and b wave, are used clinically largely for ease of identification, and actually reflect a combination of underlying retinal processes [8], [9], [24], [25]. It is possible that other parameters of the waveform may better probe the underlying physiology. The purpose of this study was to investigate novel, objective approaches to the analysis of the transient focal cone ERG [17], [26], and to compare the diagnostic capacity of these objectively determined parameters in the detection of early AMD.

Although the amplitude of the various peaks and troughs of the ERG are often variable, the inflection points are relatively consistent, particularly in the low amplitude focal cone ERG. In the literature, a small number of studies have investigated photoreceptor function by assessing the slope or gradient of the descending limb of the a-wave [27]–[30]. The gradient of the a-wave is believed to provide a cleaner marker of underlying photoreceptor function than the a-wave amplitude, whose magnitude is influenced by the ON-bipolar cell response that generates the b-wave [31], [32]. Although these studies focus on the descending limb of the a-wave, it is possible to calculate the gradient at any point along the ERG waveform using calculus to determine the 1^st^ derivative, for example the ascending and descending limbs of the b-wave. In addition by identifying the zero crossing of the 2^nd^ derivative it is also possible to determine the timing of the “peak rate of change” or maximum gradient for not only the a-wave but also the ascending and descending limbs of the b-wave. We may expect the gradient to be less susceptible to ceiling or saturation effects than conventional amplitudes and implicit times. This “peak rate of change” may also reflect different aspects of the underlying physiology compared to the implicit time and amplitude parameters conventionally measured. Although derivatives of the ERG have previously been investigated [33], as far as we are aware this approach has not previously been applied to focal ERG waveforms.

Fourier analysis and/or band pass filtering are commonly used approaches to aid the interpretation of the ERG waveform by removing high and/or low frequency noise. These techniques are used to improve the signal to noise ratio and reduce the variability of the resultant measurements. Gur & Zeevi [34] took an unconventional approach and, instead of using a Fast Fourier transform to smooth the ERG waveform, they used it to view the waveform in the frequency-domain (power spectrum). In this study they analysed 26 dark adapted full field ERG waveforms (n = 13 participants) in the frequency-domain and compared the variability of the dominant frequency to conventional measurements of b-wave amplitude and implicit time. The frequency-domain parameters demonstrated reduced variability compared to the conventional parameters. The authors suggested a number of contributory factors for this finding such as the effect of normalisation during Fourier analysis and the variability of the b-wave peak. They suggest that interpretation of ERG waveforms in the frequency-domain may prove to be beneficial for dealing with reduced signals or for the detection of certain pathologies compared to the conventional time-domain approach. This objective approach may prove particularly beneficial in focal cone ERGs where the signal is much reduced.

This paper evaluates the diagnostic ability of 4 conventional and 15 novel parameters of the focal cone ERG from the time-domain, frequency-domain and 1^st^ & 2^nd^ derivatives (see Table 1) in a cohort of patients with and without early AMD.

**Table 1:** Definitions for conventional and novel focal cone ERG parameters.

Parameter	Definitions
**Implicit times**	**Time (ms) from stimulus onset to:**
a wave *	peak of the a-wave
b wave *	peak of the b-wave
Descending a	inflection point on the descending limb of the a-wave
Ascending b	inflection point on the ascending limb of the b-wave
Descending b	inflection point on the descending limb of the b-wave
**Amplitudes**	**Potential difference (μV) between:**
a wave *	baseline and peak of a-wave
b wave *	a-wave peak and b-wave peak
**Gradients**	**Rate of change (μV/ms) at the inflection point on the:**
Descending a	descending limb of the a-wave
Ascending b	ascending limb of the b-wave
Descending b	descending limb of the b-wave
**Frequency-domain (μV)**	**Amplitude of signal at given frequency (e.g. 5 Hz).**

* - conventional electroretinogram parameters.

## Materials and Methods

### Participants

Control participants (n = 54; 69±7.7 years) and those with early AMD (n = 54; 72.9±8.2 years) were recruited from patients attending the eye clinic at the School of Optometry and Vision Sciences (Cardiff University) and the Eye Unit at the University Hospital of Wales, Cardiff. All participants had a corrected visual acuity of 0.3 LogMAR (approximately 6/12) or better, assessed using an Early Treatment of Diabetic Retinopathy Study acuity chart, and an equivalent mean spherical refractive error of less than 6 dioptres. Participants were excluded if they had secondary retinal disease, significant cataract (Lens Opacities Classification System III grade 4 or more for any criterion [35]), or narrow iridocorneal angles (grade 1, assessed by Van Herick). The study adhered to the tenets of the Declaration of Helsinki and was approved by the South East Wales Research Ethics Committee and the School of Optometry and Vision Sciences Research Ethics Committee. Each participant was given a full explanation of the procedures involved, and their written informed consent was obtained before participation in the study.

The Age-related Eye Disease Study Grading System [36] was adapted to categorise participants into either a control or early AMD group based on assessment of 37° non-stereoscopic digital retinal images (CR-DGi non-mydriatic retinal camera; Canon Inc, Lake Success, New York, USA) or 30° diameter stereo retinal images (3-DX Stereo Disc Camera; Nidek Co. Ltd., Gamagori, Japan). Early AMD was defined as the presence of soft drusen (>125 µm diameter), pigment changes, or drusenoid pigment epithelial detachment in the absence of any feature of advanced AMD (neovascular or atrophic) within a 6000 µm diameter circle centred on the fovea. Optical Coherence Tomography images were obtained for those participants undergoing non-stereoscopic imaging, to ensure the absence of any features of neovascular AMD. Control participants exhibited no features associated with AMD anywhere within the macula. Classification was carried out by two of the authors independently with discrepancies involving the consultation of the third and a majority decision taken.

### Electroretinography

One drop of Tropicamide 1.0% was instilled into both eyes of each participant, ensuring pupil dilation of at least 7 mm before retinal photography and ERG recording. For ERG recording, the earth electrode was a silver-silver chloride skin electrode applied to the midfrontal position using surgical tape (Blenderm; 3M, St. Paul, MN) after preparing the skin with abrasive gel (Nuprep; D. O. Weaver & Co., Aurora, CO), and filling the electrode cup with electrolyte electrode gel (Teca, Pleasantville, NY). A Dawson Trick Litzkow (DTL) fibre active electrode (Unimed Electrode Supplies, Surrey, UK) was positioned in the lower fornix of the test eye, and another DTL fibre positioned in the contralateral eye acted as reference. An evoked potential monitoring system (Medelec Synergy EP; Oxford Instruments Medical, Surrey, UK) was used to record all ERGs. All responses were band-pass filtered from 1 to 100 Hz and digitally averaged. An artefact reject setting allowed the exclusion of traces contaminated by blinks or eye movements.

Focal cone ERGs were recorded according to a previously described protocol [17]. In brief, an amber stimulus (λmax  = 595 nm, half-height bandwidth  = 17 nm) with an average luminance of 30 cd.m^−2^ (1190 photopic td, assuming a pupil diameter of 7 mm, and making no allowance for the Stiles' Crawford effect) subtending 20° at the eye, was presented at a temporal frequency of 5 Hz (50% duty cycle). Stimuli were generated using a miniature Ganzfeld LED stimulator. A luminance matched desensitising white square surround (30 cd.m^−2^, 118° width) was used to suppress the cones and rods of the peripheral retina. Responses were recorded on a 200 ms time base. Four traces were recorded, each consisting of an average of 100 responses (recorded in blocks of 25 to minimise blink artefacts).

### Conventional (Time-domain)

The focal ERG traces were exported and analysed using Excel (Microsoft. Redmond, WA). Each waveform was drift corrected prior to Fourier analysis following an approach described by Stroud [37]. Fourier analysis was then used to reconstruct the waveform removing all frequencies above 45 Hz, providing a “Fourier smoothed” conventional waveform in the time-domain (see Figure 1A). The positions of the a and b waves were objectively determined by identifying the local minima and maxima and confirmed by visual inspection. The amplitudes and implicit times of the a and b waves were then measured providing 4 “conventional” functional parameters (see Table 1).

### Frequency-domain

Fourier analysis was then used to convert the focal cone ERG into the frequency-domain and generate a power spectrum, the power was sampled at the first 9 harmonics of the ERG signal (f_0_  = 5 Hz) thus providing 9 functional parameters (see Figure 1B).

### Derivatives

The 1^st^ & 2^nd^ derivatives were then derived from the Fourier smoothed waveform in MatLab (Mathworks. Natick, MA). A ‘gradient method’ was applied, following an iterative paradigm with a 7 data point window, to determine the 1^st^ and 2^nd^ derivatives (see Figure 2). The location of 3 zero crossings was then objectively determined from the 2^nd^ derivative, corresponding to the inflection points on the descending limb of the a-wave, and both the ascending and descending limbs of the b-wave. The gradient (rate of change) and implicit time at each inflection point was then determined, providing 6 further functional parameters (see Table 1).

### Statistical analysis

The distribution of data for each of the 19 parameters was then assessed for normality. Where the data were not normally distributed, non-parametric statistics were applied. A student t-test indicated a small but significant difference in the age of the Control and AMD groups (p<0.05). For this reason, the data were corrected for age using linear regression analysis.

The difference between groups (AMD and Control) was assessed for each parameter using a Student t-test (two-sided), or the Mann-Whitney U test for non-normally distributed data. Receiver Operating Characteristics (ROC) were then calculated using SPSS 19 (IBM, Armonk NY) for each parameter and the area under the curve (AUC) used to assess diagnostic ability.

For all parameters where a statistically significant difference (p<0.05) between groups was identified, a discriminant analysis was performed using logistic regression (following a forward stepwise likelihood ratio paradigm) in SPSS 19 (IBM, Armonk NY) to identify the best (or best combination of) parameter(s) that predict the presence of early AMD. Receiver Operating Characteristics (ROC) curves were then constructed on the discriminant analysis model.

Using the method described by Hanley and McNeil [38], ROC curves were compared to determine whether any of the new parameters, or the discriminant analysis model, provided a statistically better diagnostic potential than the best conventional ERG parameter.

## Results

**Figure 3:**
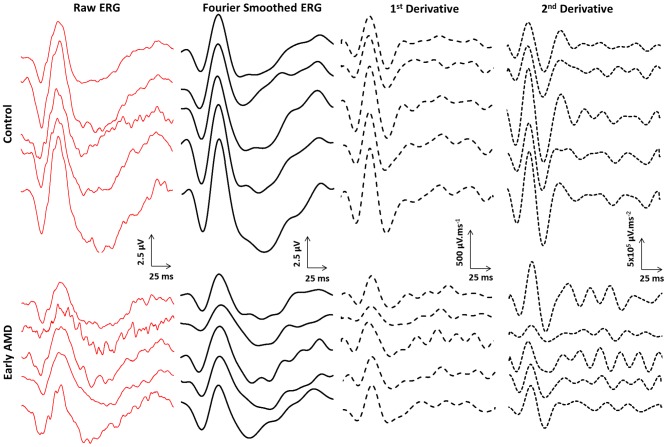
Focal cone ERG waveforms & derivatives. Representative raw, fourier smoothed, 1^st^ derivative and 2^nd^ derivative waveforms of the focal cone ERG are shown for 10 study participants. The waveforms shown were recorded from five healthy participants (TOP) and five early AMD participants (BOTTOM).

Focal cone ERGs were obtained successfully from all participants. Raw traces for 5 controls and 5 participants with early AMD are shown in Figure 3. There was a significantly reduced visual acuity in the early AMD group (mean logMAR 0.15±0.15) compared to the Control group (mean logMAR 0.0±0.09, p<0.05). Lens Opacities Classification System III [35] grading of lenticular opacities did not reveal a significant difference between group for any of the 4 grading criteria. Mean grades were 1.9±1.1 and 1.9±1.0 for Nuclear Opalescence, 1.9±1.0 and 1.9±1.0 for Nuclear Colour, 0.9±1.0 and 0.7±0.9 for Cortical Cataract and 0.3±0.6 and 0.4±0.6 for Posterior Sub-capsular in the early AMD and Control groups respectively.

### Conventional (Time-domain)

**Figure 4:**
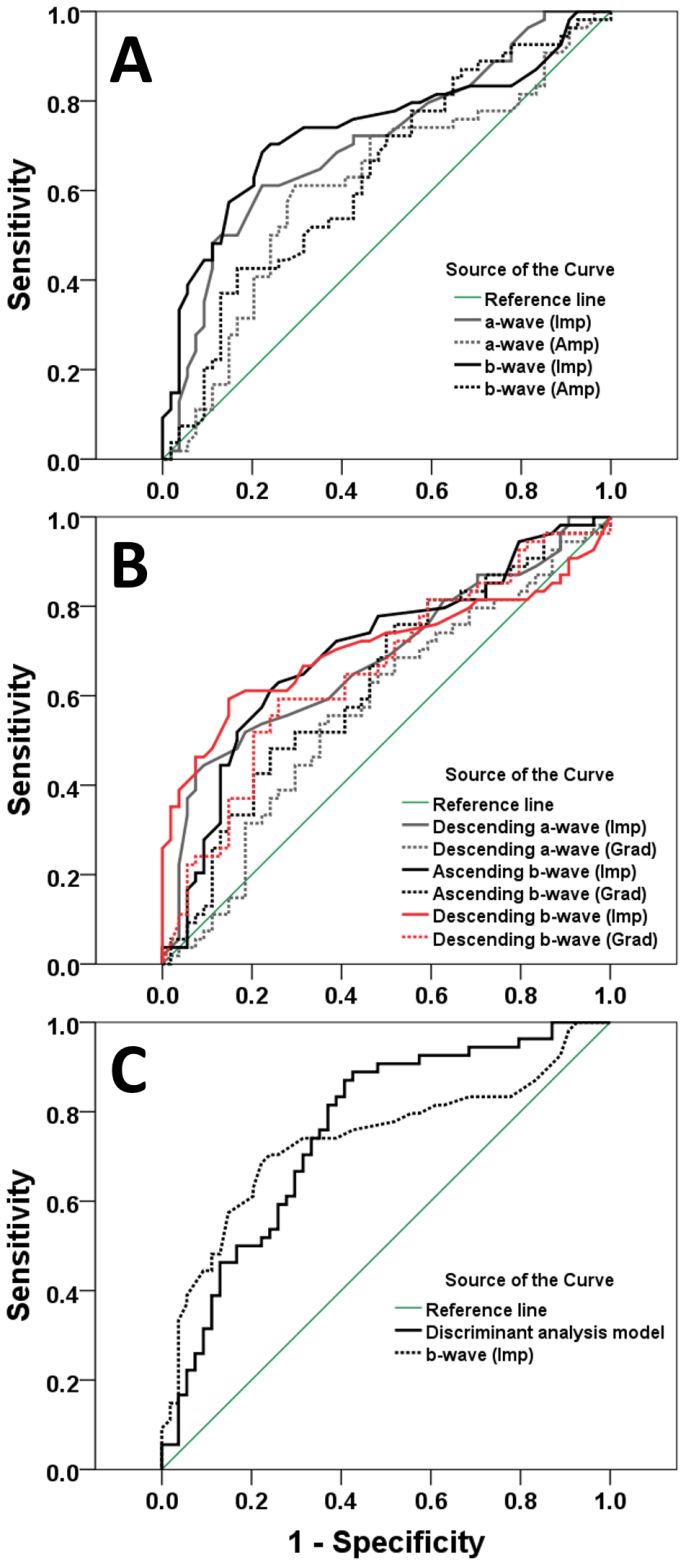
Receiver operating characteristic curves for study parameters. (A) Conventional parameters, (B) 1^st^ & 2^nd^ derivative parameters, (C) Discriminant analysis (Key used to denote individual parameters). Each plot shows the sensitivity of the parameter to early AMD against the false detection rate (1 - specificity) for all study participants (n = 108), a greater area under the curve (AUC) indicates better discriminatory ability. Abbreviations - Imp  =  implicit time; Amp  =  amplitude; Grad  =  gradient

Typical focal cone ERG traces, with frequencies above 45 Hz removed, are shown for 5 controls and 5 participants with early AMD (see Figure 3). Generally, the participants with early AMD had smaller amplitudes and delayed implicit times for both the a and b waves compared to participants in the control group. Delays in the mean a and b wave implicit times of 1.43 and 2.79 ms, respectively, were found to be statistically significant (p<0.001; see Table 2). ROC analysis produced an AUC for a- and b-wave implicit times of 0.71 and 0.74, respectively, demonstrating good diagnostic potential (see Figure 4A). The reductions in mean a and b wave amplitudes in the AMD group of 0.34 and 0.74 µV were both statistically significant (p<0.05), however ROC analysis suggested a reduced diagnostic potential compared with their equivalent implicit times, returning AUC values of 0.62 and 0.64, respectively (see Figure 4A).

**Table 2:** Focal cone ERG parameters for Control and AMD groups showing discriminatory and diagnostic ability.

Parameter	Control group		AMD group			
	μ	σ	μ	σ	p-value	AUC
**Implicit time (ms)**						
a wave	23.50	*2.03*	24.93	*1.94*	<0.001*	0.71
b wave	43.63	*2.26*	46.42	*3.72*	<0.001*	0.74
Descending a	16.60	*1.61*	17.79	*1.86*	<0.001*	0.68
Ascending b	33.66	*2.11*	35.08	*2.26*	<0.001*	0.70
Descending b	53.24	*2.66*	57.62	*5.26*	<0.001*	0.71
**Amplitude (μV)**						
a wave	−1.93	*0.83*	−1.58	*0.85*	0.037*	0.62
b wave	4.64	*1.67*	3.90	*1.61*	0.021*	0.64
**Gradient (μV/ms)**						
Descending a	−186	*72*	−163	*68*	0.097	0.57
Ascending b	370	*131*	299	*127*	0.005*	0.63
Descending b	−274	*89*	−233	*99*	0.033*	0.66
**Frequency-domain (μV)**						
5 Hz	1.05	*0.51*	0.98	*0.43*	0.454	0.54
10 Hz	0.98	*0.36*	0.94	*0.39*	0.603	0.55
15 Hz	0.70	*0.25*	0.64	*0.27*	0.223	0.59
20 Hz	0.56	*0.20*	0.48	*0.22*	0.070	0.61
25 Hz	0.54	*0.20*	0.43	*0.22*	0.006*	0.68
30 Hz	0.27	*0.11*	0.21	*0.10*	0.003*	0.68
35 Hz	0.23	*0.10*	0.19	*0.11*	0.051	0.64
40 Hz	0.11	*0.06*	0.10	*0.05*	0.186	0.57
45 Hz	0.08	*0.04*	0.08	*0.05*	0.884	0.51

μ - mean

σ - standard deviation

* - statistically significant (p<0.05)

AUC - Area under the curve for Receiver operating characteristics

### Frequency-domain

**Figure 5:**
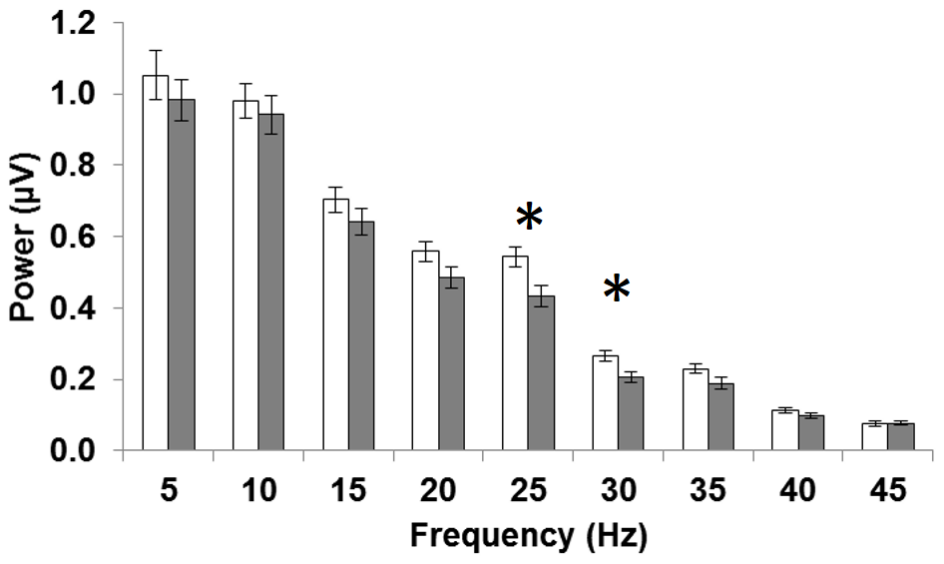
Group average frequency-domain power spectrums. The mean focal cone ERG signal for all control (White) and early AMD (Grey) participants are shown in the frequency-domain (power spectrum) at the fundamental frequency (f_0_ = 5 Hz) and its harmonics up to 45 Hz. Error bars show standard error whilst stars (*) denote statistical difference between groups at the p = 0.05 significance level.

The frequency-domain analysis produced a power spectrum peaking at the fundamental and reducing with increasing frequency (see Figure 5). Focal cone ERGs in the early AMD group showed a mean reduction in power across all 9 frequencies assessed, an outcome that might be expected given the reduction in mean amplitude of both the a and b waves. However, these differences were only statistically significant for the 5^th^ (25 Hz) and 6^th^ (30 Hz) harmonics (see Table 2). When the diagnostic potential of these parameters was assessed using ROC analysis, they both returned AUC values of 0.68, indicating only moderate diagnostic value compared to the best parameter evaluated in this study (b-wave implicit time) with an AUC of 0.74.

### Derivatives

The 1^st^ & 2^nd^ derivatives were used to identify the timing and magnitude of the “peak rate of change” or point of maximum gradient for the descending limb of the a-wave, and both the ascending and descending limb of the b-wave. Figure 3 shows representative data from 5 control and 5 early AMD participants. This analysis showed that the gradient at all 3 points was reduced in the AMD group compared to controls, and the corresponding implicit time was likewise delayed. The gradient however was only significantly decreased (p<0.05) on the ascending and descending limb of the b wave, with changes of 71.06 and 40.71 µV.ms^−1^, respectively. In contrast, the time to the “peak rate of change” was found to be significantly prolonged in all 3 cases, with delays of 1.19, 1.42 and 4.38 ms (descending a, ascending b, and descending b wave limbs). At each of the 3 inflection points assessed, the implicit times provided greater diagnostic potential than their equivalent gradient parameters (see Table 2). Receiver operating characteristic analysis revealed that the AUC for the implicit times of the descending a, ascending b and descending b inflection points were 0.68, 0.70 and 0.71 respectively, compared to 0.66 for the best performing gradient parameter (the descending b-wave inflection point) (see Figure 4B).

Finally, the AUC for the conventional b-wave implicit time was then compared to the conventional a-wave implicit time, and the descending a, ascending b and descending b times to “peak rate of change”, which were of a similar magnitude (0.71, 0.68, 0.70 & 0.71 respectively). Z values of 0.807, 1.307, 1.396 and 1.136 were returned respectively, none of which reached the 95% significance level (z>1.96), indicating that there was no significant difference in diagnostic capacity between these parameters. Therefore, when considered in terms of potential diagnostic ability, the implicit times of the inflection points consistently provided the best AUC, and were equivalent to the best performing conventional parameter, namely the b-wave implicit time.

In total, nineteen comparisons of focal cone ERG parameters were made between the control and early AMD group as part of this study. It could be expected that the null hypothesis would be wrongly rejected in 1 comparison on the basis of chance alone (i.e. a type I error). As the nature of this analysis was exploratory rather than confirmatory, the use of a conservative multiple testing correction, such as Bonferroni, was not appropriate as it would be expected to increase type 2 errors. Furthermore, the majority of the 9 frequency-domain parameters tested were correlated (mean 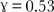 across all harmonics, Pearson correlation coefficient), in such cases the risk of a type I error decreases with multiple testing [39].

### Discriminant Analysis

In addition to evaluating each parameter individually, discriminant analysis using logistical regression was performed (IBM SPSS 19, Armonk NY) on all parameters that demonstrated a statistically significant difference between groups (see Table 2). The discriminant analysis identified the b-wave implicit time and amplitude plus the power of the 5^th^ harmonic (25 Hz) as the strongest predictor variables. When these parameters were combined in a model, the analysis returned an optimal sensitivity and specificity of 82.4 and 77.6% respectively for discrimination between the control and AMD groups in this study.

The discriminant analysis model produced an improved AUC of 0.76 compared to the highest AUC for an individual parameter of 0.74, attributable to the b-wave implicit time (see Figure 4C). However the difference in AUC was not found to be statistically different (z = 0.380 <1.96), indicating that the combined predictors do not provide a significant diagnostic advantage over the b-wave implicit time alone.

## Discussion

In this study, we demonstrated two novel approaches to the analysis of the focal cone ERG, and compared the diagnostic capacity of the parameters to a more conventional approach based on peak-to-trough measurements. For the conventional and novel analytic approaches, the timing based parameters showed the greatest ability to identify people with early AMD. The diagnostic accuracy (described by the AUC of the ROC analysis) was comparable between the conventional parameters and the derivative analysis, whilst a discriminant analysis model provided a modest improvement over any individual parameter alone.

The conventional parameters of the focal cone ERG waveform were comparable to those previously reported using this technique in participants with early AMD [17]. The a and b wave implicit times were significantly delayed whilst the amplitudes were significantly reduced compared to controls. Overall, focal cone ERG parameters based on implicit times appeared to provide the greatest sensitivity to disease, both for the time to peak and the newly evaluated time to inflection point (“peak rate of change”). This is, perhaps, unsurprising, as implicit time has been shown to be less variable than amplitude. For example, the position and type of electrodes used to record the ERG have been shown to significantly affect the amplitude of the a-wave, b-wave and Photopic Negative Response (PhNR) whilst, in contrast, the implicit times of these parameters have been shown to be far more robust [40], [41]. Inter-individual variations in anatomical features are likely to influence the placement of skin electrodes, whilst a combination of blinks and eye movements during testing may change active electrode position and, consequently, the measured amplitude of the ERG waveform. Furthermore, variations in axial length and fundus pigmentation between individuals have also been shown to impact upon ERG amplitude [42], [43]. This inherent variability in all amplitude measures will ultimately limit sensitivity.

The parameters of 1^st^ & 2^nd^ derivatives used in this study have not, to our knowledge, previously been applied clinically. The parameters based on implicit time, both conventional and those measured from the 2^nd^ derivatives, proved to be the most sensitive discriminators. The objectivity and low variability of these implicit time parameters, suggest that they are promising candidates for use as markers of retinal function in early AMD, or other retinal pathology, in future investigations. Furthermore, given the linear nature of the ERG, we expect ‘sick’ components to cumulatively add to delays in the response. The observation that all implicit times were equally affected in this study suggests that the delay originates in a distal part of the retina, most probably the photoreceptors. We hypothesise that pathology affecting more proximal retina would result in delayed implicit times for later components only. The analysis of derivatives offers the opportunity to sample the implicit time of the ERG at points other than the peaks or troughs of the a and b waves (i.e. providing greater temporal resolution).

In this study the frequency-domain of the focal cone ERG was analysed for a 200 ms window. Visually, the resulting power spectra all demonstrated a peak skewed towards the low frequencies, consistent with previously published data using a similar sized window and a bright photopic stimulus for full field ERGs [44]. However, Gur & Zeevi [34] suggest employing a smaller window (i.e. first 55 ms of the response) comprising the majority of the a and b-wave contribution but less susceptible to contamination, for example by components attributable to eye movements. Furthermore, given that the a and b wave components are known to be affected in AMD [17], [45], this approach could potentially be more sensitive to disease related change in these patients. The frequency-domain analysis used in this study also limited the power spectrum to a range of between 5 to 45 Hz, with the intention to remove content known to be attributable to inner retinal function, specifically the Oscillatory Potentials [46]. Whilst this should retain components known to originate in the outer retina (i.e. the a and b-wave components), it is not impossible that high frequency contributions could also be affected in early AMD.

Although the power spectrum appeared to provide limited diagnostic value in this study, the mathematical nature of this approach is particularly suited to automated analysis, removing a number of the limitations and subjective aspects of ERG waveform interpretation [34]. This attribute could be valuable in developing a robust ERG based clinical test for use beyond the laboratory. It should also be noted that this approach has not been extensively studied, consequently a greater understanding of the retinal origins of the power distribution and the underling physiological process involved may allow optimisation of test parameters and have the potential to provide new insight into retinal diseases [34]. Whilst it would be possible to speculate on the precise retinal origins and/or the physiological processes contributing to all the parameters described, this was beyond the scope of this investigation.

Finally, the discriminant analysis showed that additional diagnostic value can be achieved by combining the novel and conventional ERG parameters. This is particularly important finding clinically, as it shows that additional diagnostic potential can be achieved without the need for additional data acquisition or testing.

In conclusion, the novel analytical techniques evaluated in this manuscript provide potentially greater objectivity whilst demonstrating sensitivity comparable to the conventional a and b waves in early AMD. If focal ERG techniques are to be used in the monitoring of AMD and of visual function post treatment, well-defined, reproducible and objective analysis will be of key importance. We believe these techniques could therefore prove valuable in the investigation, detection and monitoring of early AMD in the future.
